# USE OF COMPLEMENTARY THERAPIES BY SEXUAL MINORITY OLDER ADULTS: FINDINGS FROM THE NATIONAL HEALTH INTERVIEW SURVEY

**DOI:** 10.1093/geroni/igz038.536

**Published:** 2019-11-08

**Authors:** Joel G Anderson, Jason D Flatt, Jennifer Jabson, Whitney Wharton

**Affiliations:** 1 University of Tennessee, Knoxville, Knoxville, Tennessee, United States; 2 University of California, San Francisco, San Francisco, California, United States; 3 Emory University, Atlanta, Georgia, United States

## Abstract

Sexual minority (lesbian, gay, bisexual; LGB) older adults age 50+ experience a higher prevalence of chronic disease and disability, as well as a poorer physical and mental health status. Many adults use complementary and integrative therapies, particularly mind-body therapies, as health-enhancing approaches and to support wellbeing. However, no study to date has examined the use of mind-body therapies among sexual minority older adults. We examined data from the 2017 National Health Interview Survey to determine the use of mind-body therapies by sexual minority older adults (aged 50+), as well as the influence of health and wellbeing characteristics on mind-body therapy use, compared with their non-LGB counterparts. Sexual minority older adults overall reported higher usage (36%) of mind-body therapies compared with their non-LGB counterparts (22%), with lesbians reporting the highest use (41%). Sexual minority identity was a significant predictor of mind-body therapy use, with LGB adults roughly two times more likely to use a mind-body therapy after controlling for chronic disease status and other wellbeing measures. Future research is needed to explore the reasons sexual minority older adults use complementary and integrative therapies, as well as potential development of mind-body interventions targeted toward this population to address stress and quality of life.

